# Specific Epiblast Loss and Hypoblast Impairment in Cattle Embryos Sensitized to Survival Signalling by Ubiquitous Overexpression of the Proapoptotic Gene BAD

**DOI:** 10.1371/journal.pone.0096843

**Published:** 2014-05-07

**Authors:** Jessica van Leeuwen, Debra K. Berg, Craig S. Smith, David N. Wells, Peter L. Pfeffer

**Affiliations:** 1 Animal Productivity, AgResearch, Hamilton, Waikato, New Zealand; 2 Department of Biological Sciences, University of Waikato, Hamilton, Waikato, New Zealand; 3 School of Medicine, University of Notre Dame, Sydney, New South Wales, Australia; Instituto de Medicina Molecular, Portugal

## Abstract

Early embryonic lethality is common, particularly in dairy cattle. We made cattle embryos more sensitive to environmental stressors by raising the threshold of embryo survival signaling required to overcome the deleterious effects of overexpressing the proapoptotic protein BAD. Two primary fibroblast cell lines expressing *BAD* and exhibiting increased sensitivity to stress-induced apoptosis were used to generate transgenic Day13/14 *BAD* embryos. Transgenic embryos were normal in terms of retrieval rates, average embryo length or expression levels of the trophectoderm marker *ASCL2.* However both lines of *BAD*-tg embryos lost the embryonic disc and thus the entire epiblast lineage at significantly greater frequencies than either co-transferrred IVP controls or *LacZ*-tg embryos. Embryos without epiblast still contained the second ICM-derived lineage, the hypopblast, albeit frequently in an impaired state, as shown by reduced expression of the hypoblast markers *GATA4* and *FIBRONECTIN*. This indicates a gradient of sensitivity (epiblast > hypoblast > TE) to BAD overexpression. We postulate that the greater sensitivity of specifically the epiblast lineage that we have seen in our transgenic model, reflects an inherent greater susceptibility of this lineage to environmental stress and may underlie the epiblast-specific death seen in phantom pregnancies.

## Introduction

The study of early embryogenesis is of particular relevance to the dairy cattle industry as embryo mortality is high and causes significant financial losses [1]. A high rate of embryo loss may not be so surprising considering the number of critical events taking place during the first two weeks of development [2], [3]. Four days after fertilization, cattle embryos reach the 16-cell stage with compaction generally occurring on Day 5 with the formation of distinct inner and outer cells [4]. A day later the embryo, consisting of approximately a hundred cells, undergoes blastulation as seen by the formation of an asymmetrically located cavity. At this stage the mammalian embryo is known as a blastocyst with the outer “trophectoderm” (TE) cells giving rise to the trophoblast (placenta) and the inner cell mass (ICM) cells the rest of the conceptus. Cattle embryos hatch from their surrounding proteinaceous capsule, the zona pellucida, after Day 8. By this time, the ICM has begun to differentiate into two cell populations: the epiblast and the underlying hypoblast which faces the blastocyst cavity [5]. During the following two days, the hypoblast cells divide and migrate along the basal surface of the TE to line the blastocyst cavity. The epiblast and underlying hypoblast are termed the embryonic disc once the trophectoderm overlying the epiblast (Rauber's layer) disappears, at around Day 12. During the next few days the epiblast flattens into a two-layered structure with outer epiblast cells connecting via tight junctions, characteristic of an epithelium. Embryonic patterning and mesoderm formation starts at Day 14, when cells are seen to accumulate at one pole of the embryonic disc [2], [3]. Concurrently, the trophectoderm cells proliferate rapidly, leading to the rapid elongation of the conceptus. Trophectoderm cells begin secreting the ruminant pregnancy recognition factor Interferon-tau from Day 14. Interferon-tau, by repressing the upregulation of oxytocin receptor transcription in the endometrium, counteracts uterine prostaglandin F2α secretion which, if unchecked, would otherwise lead to corpus luteum degeneration and termination of pregnancy [6].

In vivo, every eighth cattle embryo dies during the second week of embryogenesis [7], whereas a quarter of embryos die during this period when previously stressed by being cultured in vitro to the Day 7 blastocyst stage [8]. Additionally, a quarter of these embryos recovered on Day 14 lack an embryonic disc, thus being destined to die [8]. That mammalian embryos can be cultured at all until the blastocyst stage in defined relatively simple media indicates that they are autopoietic in their requirements. The beneficial effects of group culture has been linked to the (autocrine) release of survival factors into the medium. Hence, growing embryos in large volumes of media, thereby diluting out any secreted factors, results in reduced viability [9], [10]. The difference in post-blastocyst survival between embryos grown in vitro and in vivo means, however, that autocrine signaling within the culturing systems does not fully recapitulate the conditions of the reproductive tract. The role of maternal survival signals is undisputed at later stages, since cattle embryos grown in culture past Day 7 rapidly lose their viability [11], [12]. Some of the maternal factors originate from endometrial gland secretions as embryo development is severely impaired by Day 14 in “uterine gland knock-out” sheep, which lack endometrial glands [13].

Most of the autocrine factors secreted by early mammalian embryos and whose exogenous addition to culture media have been shown to improve survival (the embryotrophins PAF, insulin, IGF1, CSF2), act via a phosphatidylinositol-3 kinase (PI3K) dependent survival signaling pathway [14]–[16]. This pathway leads to (i) an AKT-mediated decrease in activity of proapoptotic effectors such as BAD and BAX (by TP53 inhibition) and (ii), via an intracellular rise in calcium concentration, to increased transcription of the anti-apoptotic *BCL2* gene [17]. Embryotrophins can display differential and opposing effects in cells of alternate lineages. For example, IGF1 is required for trophectoderm survival in the mouse and in cattle [18], [19], whereas CSF2 specifically increases the number of ICM cells in cattle blastocysts [20].

This raises the question as to which early lineages are most susceptible to changes in the environment. The answer has important consequences for attempts to improve embryo viability, particularly after in vitro culture. We attempted here to increase the overall sensitivity of the embryo to its environment by overexpressing a weak proapoptotic gene in cattle preimplantation embryos. We decided to use BAD for this purpose. BAD is a BH3-domain-only member of the BCL2 family of cell death regulators. When not phosphorylated, BAD binds to and neutralizes anti-apoptotic BCL2 proteins [21], [22]. This prevents BCL2 from inhibiting the proapoptotic BAX and BAK proteins which mediate all death stimuli that act through the intrinsic pathway of apoptosis [23]. BAD appears to be a “weak” proapoptotic gene, as loss of function mouse mutants display minimal defects [24]. Its main role is to modulate the response of cells to proapoptotic stimuli such as heat shock, starvation and radiation induced damage. This is achieved predominantly via regulation of its activity through phosphorylation. Constitutively dephosphorylated BAD sensitizes cells to proapoptotic stimuli [25]. However, BAD phosphorylation, induced by numerous trophic survival signals, raises the threshold level at which mitochondria release Cytochrome c to induce apoptosis in response to death signals [25], [26]. We show here that *BAD* messenger RNA overexpression, expected to enhance the dependence of cells on trophic survival signals, resulted in very specific cell lineage dependent cell death.

## Methods and Materials

### Ethics statement

Animal procedures were conducted under the approval of the Ruakura Animal Ethics Committee (Permit R.A.E.C. 11183). This permit lists the efforts made to minimize animal suffering.

### Generation of *BAD*-overexpressing cell lines

Bovine *BAD* (NM_001035459) was PCR amplified using *Xho1* restriction site-flanked primers 5′GTG*CTCGAG*CATGTTCCAGATCCCAGA and 5′GTG*CTCGAG*CGGTTGGGAGCTCCGGTT using cDNA from a Day 20 cattle embryo as a template and Expand High Fidelity PCR system polymerase (Roche, Auckland, NZ) with 10 cycles of: 94°C for 15 sec, 56°C for 30 sec, 72°C for 45 sec, followed by 20 cycles of: 94°C for 15 sec, 56°C for 30 sec, 72°C for 45 seconds plus 5 seconds each cycle and a final elongation at 72°C for 7 min. The amplicon was purified using the DNA Clean and Concentrator kit (Zymo Research, Irvine, CA), digested with *Xho1* (Roche, Auckland, NZ), and purified from a 1% agarose gel using the WIZARD SV gel and PCR clean-up system (Promega, Auckland, NZ). The vector, pPyCAGiP [27], kindly supplied by H. Niwa, was *XhoI* digested, Calf Intestinal Phosphatase (Roche) treated and gel purified. Vector and insert were ligated at equimolar ratios using Mighty Mix (Takara) to create *pCAG-BADiPuro*. The plasmid clone used here was verified by sequencing. Primary bovine female embryonic fibroblast cells (EF5) were stably transfected with *pCAG-BADiPuro* using Lipofectamine-2000 according to the manufacturer's instructions (Life Technologies, Auckland, NZ). After puromycin selection, individual colonies were picked, expanded and transgenic *BAD* expression measured by quantitative PCR. Generation of the *pCAG-LacZiPuro* construct and cell lines has been previously described [28]. Before use in nuclear transfer, cell lines were karyotyped according to standard procedures.

### Apoptosis induction assay

Cells from each cell line were plated in quadruplicate at 3×10^5^ cells/well in 6 well plates and grown for two days. Two wells of each cell line were exposed to 90 mJ/cm^2^ 254 nm UV radiation in a UV Stratalinker 1800 (Agilent Technologies, Santa Clara, CA). Cells were harvested 20 hours later using Tryple (Life Technologies), rinsed in PBS and Caspase activity measured using the EnzCheck Caspase-3 Assay Kit #1 as per instructions (Molecular Probes, Eugene, USA).

### Generation of NT and IVF Embryos

Somatic cell nuclear transfer (NT) embryos were generated as described in detail [29]. Oocytes from the same pool of ovaries were used as cytoplasts for zona-free somatic cell nuclear transfer (NT) and zona-free single culture IVF control embryos. In vitro fertilization was as described [30] with the following modifications. The zona pellucida was removed from IVF generated zygotes with protease digestion (pronase *Strep. Griseus*, 0.5% in HSOF supplemented with 1 mg/ml PVA). The zona-free zygotes were singly cultured in 5 µl drops under oil as described for NT generated embryos [29]. Seven days after single culture of IVF or NT generated embryos, groups of grade 1 and 2 early to expanded blastocysts were selected for transfer by morphological evaluation [31]. Grading and selection of blastocysts were completed by the same experienced embryologist throughout the entire data collection period to reduce variability. The number of blastocysts transferred per recipient was 10 NT and 5 grade and stage-matched IVP into the ipsilateral uterine horn for line two. For line one, 11 NT and 10 IVP were transferred into opposite horns as described [32]. Recipients consisted of six multiparous non-lactating dairy cows that had been tested for their suitability as optimal recipients by repeated transfer and recovery of embryos. For the control NT experiment using CAG-LacZ transgenic cells, 2–7 embryos were transferred into untested recipients. Recipient cows were synchronized using a single intra-vaginal progesterone-releasing device, selected for estrus and embryos transferred transcervically as described [32]. Co-transferred embryos were recovered by non-surgical flushing at 6 or 7 days post-transfer, corresponding to a gestational age of 13 or 14 days as described in [8]. CAG-LacZ embryos were recovered at age E14 and E15. The same operator transferred and recovered the embryos.

### Embryo analyses

Embryos were identified by stereomicroscopy, their origin recorded, total length measured using graduated eyepieces and examined for the presence of an embryonic disc/epiblast. Embryos were then cut into several fragments for use in genotyping and gene expression analyses. For the identification of transgenic embryos, an embryo fragment was PCR-genotyped, after a 2 h digestion at 55°C with shaking at 900 rpm in 30 µl proteinase K buffer [33], using primers CAG-BAD (Table 1) and 0.25 µl of centrifuged (16000 g, 10 min) lysate.

**Table 1:** PCR primers.

*Gene*	*Forward*	*Reverse*
*ASCL2*	CTCGACTTCTCCAGCTGGTTA	AGTGGAAGGTCTCTGCGGACA
*BAD^a^*	TTATGCAAAACGAGGCTCGG	GGGTTAATCTCGGCTCGCAA
*CAG-BAD^b^*	CCGACCGAAAGGAGCGCACGA	CTCATTTTATTAGGAAAGGACAG
HK (3):		
*Cyclophilin*	GCATACAGGTCCTGGCATCT	TCTCCTGGGCTACAGAAGGA
*GAPDH*	CTCCCAACGTGTCTGTTGTG	TGAGCTTGACAAAGTGGTCG
*HPRT*	GCCGACCTGTTGGATTACAT	ACACTTCGAGGGGTCCTTTT

*a* These primers lie in the 3′ UTR of cattle *BAD*. As this region was not cloned into *pCAG-BAD*, they amplify only the endogenous *BAD*.

*b* Ectopic *BAD* expression as well as genotyping were performed with these primers (163 bp amplimer) which lie in the 3′ UTR of the pCAG vector.

For β-Galactosidase staining, embryos were washed in PBS, then fixed for 15 min on ice in 0.2% glutaraldehyde, 0.1M phosphate buffer ( =  “PO_4_”; pH 7.4), 5 mM EGTA, 2 mM MgCl_2_, followed by three RT 5 min washes in 0.1M PO_4_, 2 mM MgCl_2_, 0.01% deoxycholate, 0.02% Nonidet P-40 ( =  “WASH”). Staining was done at 30°C for several hours in WASH containing 20 mM Tris-HCl pH 7.3, 5 mM K_3_(Fe(CN)_6_), 5 mM K_4_(Fe(CN)_6_) and 1 mg/ml X-galactosidase.

### Expression analyses

RNA isolation, spike addition, reverse transcription, real-time PCR and quantification procedures were performed as detailed previously [34], with the following modifications. The mini-column step was replaced with an ethanol precipitation and wash. Real time PCR was done on a Corbett Rotorgene 6000 (Qiagen, Bio-Strategy, Auckland, New Zealand) with SYBR ExTaq Mix (Takara Bio Inc., Shiga, Japan) with 3 min initial denaturation, followed by 40 cycles of 95°C for 10 sec, 60°C for 25 sec. For primer details see Table 1. We quantified transcripts relative to the geometric mean using three housekeepers (*HK*) while normalizing for different amplification efficiencies, *a*, as follows: expression level of gene of interest (*goi*)  =  [*a*_goi∧_(-Ct_goi_)]/([*a*_HK1∧_(-Ct_HK1_) × *a*_HK2∧_(-Ct_HK2_) × *a*_HK3∧_(-Ct_HK3_)]_∧_(1/3)), where Ct represents the number of cycles required to reach a constant threshold level of fluorescence and the term *a*_x_∧(-Ct_x_) is equal to the starting concentration of gene X (which is the variable to be measured), times a constant that depends on the threshold level. Each sample was measured in triplicate, one measurement being of a twofold dilution. Samples not showing half the copy number ± 50% when diluted twofold, were deemed to lie outside the linear range and discarded. A no template control, RT- control and dissociation curve analysis were included in each real-time run.

### Statistical analysis

The significance of the differences in embryo culture, number of embryos retrieved and embryos retrieved that contain an epiblast or not was calculated for each line of BAD expressing embryos and the corresponding co-transferred IVP controls using Fisher's exact test (Tables 2–4). Additionally, logistic regression analyses using modeling of binomial distributions were used to examine the significance of the proportion of embryos with epiblast, using GenStat statistical software (VSN International, Oxford, UK). The natural logarithm of embryo length and *ASCL2* expression were analyzed for recipient cow, embryo age, and embryo genotype (BAD-transgenic versus IVP) effects using REML in GenStat, specifying transfer batch as a random effect to take account of the structure of the experiment.

**Table 2:** In vitro development to Day 7 of zona free nuclear transfer transgenic and singly cultured control IVP embryos.

	*Eggs*	*2-Cell*	*Early dev (%)^a^*	*P^c^*	*Late dev (%)^b^*	*P^c^*
pCag-BAD (line 1)*^d^*	158	154	121 (79%)	5.0E-13	48 (40%)	0.25
pCag-BAD (line 2)*^d^*	157	151	106 (70%)	3.5E-08	45 (42%)	0.45
IVP*^d^*	210	184	73 (40%)		36 (49%)	

*a* Number and percentage of cleaved embryos developing to at least morula stages;

*b* Number of Grade 1 or 2 blastocysts and as a percentage of those embryos having developed to at least morula stages;

*c* Significance of difference between tg lines and IVP embryos as determined by Fisher's Exact test.

*d* These embryos were grown concurrently.

**Table 3:** No difference in the proportions of BAD overexpressing and IVP co-transferred embryos recovered on Day 13/14.

	*Embryos transferred*	*Embryos retrieved (%)*	*P*
pCAG-BAD (line 1)	33	18 (54%)	0.37
IVP cotransferred	30	12 (40%)	
pCAG-BAD (line 2)	30	14 (47%)	0.92
IVP cotransferred	15	8 (53%)	

**Table 4:** Significantly fewer *BAD* overexpressing embryos retrieved on Days 13/14 contain an epiblast compared to co-transferred IVP controls.

	*Embryos retrieved*	*Embryos with Epiblasts (%)*	*P^a^*
pCAG-BAD (line 1)	18	5 (28%)	0.029
IVP cotransferred	12	9 (75%)	
pCAG-BAD (line 2)	14	0 (0%)	0.00075
IVP cotransferred	8	6 (75%)	

*a* Using Fisher's Exact Test. A general linear mixed model with embryonic age, recipient and genotype as variables resulted in significant difference for genotype (BAD vs IVP) of P = 0.017.

## Results

**Figure 1:**
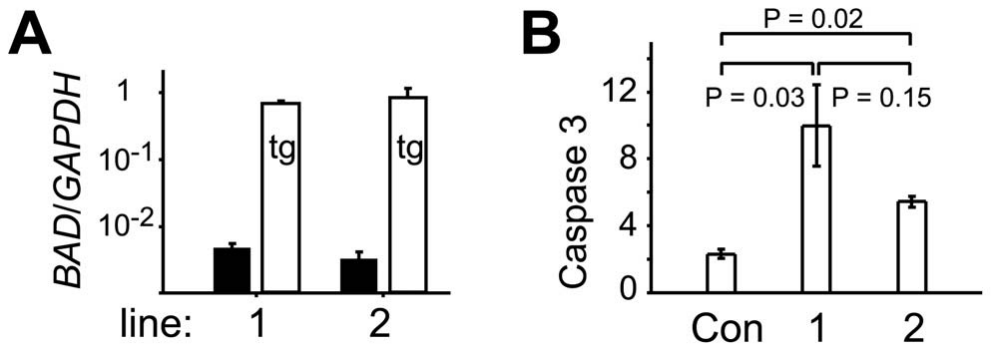
BAD expression and resistance to apoptosis in cattle primary fibroblast cell lines. A. Real-time RT-PCR measurements of endogenous (black) and *pCAG-BAD*-derived transgenic *BAD* (white) levels in two stably transfected cattle EF5 cell lines. Expression levels have been normalized to *GAPDH*. B. Apoptosis assay measuring the activation of the cell death effector Caspase 3 after irradiation with UV light. The ratio of irradiated to non-irradiated cells is shown. Con refers to control EF5 cells, 1 and 2 to the two lines of *pCAG-BAD*-transgenic cells used throughout, error bars are s.e.m., P values derived from t-test on log ratios of treatment corrected for background readings of corresponding controls.

To sensitize embryos to environmental stress and increase their reliance on trophic survival signals secreted either by themselves or, after embryonic day (Day) 7, by their maternal environment, we genetically modified embryos to overexpress the proapoptotic *BAD* gene. Cattle *BAD* was isolated by PCR from a peri-implantation embryo and inserted downstream of the chimeric **C**MV/Chick-β-**a**ctin/Rabbit-β-**g**lobin enhancer-promoter-intron module (“*CAG*”, [27]) which we have previously shown to confer strong ubiquitous expression in cattle preimplantation embryos [28]. The presence of an IRES-puromycin cassette 3′ to the *BAD* gene allowed for selection of cattle primary fibroblast (EF5) cells stably transfected with the construct. Two lines expressing *BAD* at 100-fold excess of endogenous levels (Fig. 1A) were chosen. Transcript levels of *BAD* approached that of the strongly expressed *GAPDH* housekeeper gene (Fig. 1A). To determine whether the elevated levels of BAD resulted in increased sensitivity of these cells to apoptosis, we subjected them to UV radiation-induced damage which is expected to lead to cell death via the intrinsic pathway [35]. The initiation of apoptosis is characterized by the activation of caspases 3 and 7 [35]. We observed a twofold activation in caspases 3 and 7 within 20 hours of irradiating EF5 cells (Fig. 1B). In contrast, *BAD* overexpressing EF5 lines 1 and 2 showed a ten- and fivefold increase in cell death, indicative of a proapoptotic sensitization (Fig. 1B).

We next determined how *BAD* overexpression affected embryonic development to the blastocyst stage. Control embryos and BAD-transgenic (tg) embryos created by somatic cell nuclear transfer with either line 1 or 2 were grown in single culture. Single culture is more stressful to embryos than group culture because embryotrophic signals are diluted, resulting in lower developmental rates [10], [17]. Interestingly, in spite of the stringent culture conditions, development was not worse in both lines of *BAD*-tg embryos compared to IVP embryos cultured in parallel (Table 2). More specifically, development to at least compact morula stages was nearly twice as high in the transgenic embryos, whereas development from compact morula to transferable grade embryos was slightly, but not significantly, lower in *BAD* overexpressing embryos (Table 2). However, the relatively high early developmental rates of the transgenic embryos is a non-specific nuclear transfer effect and the presently observed rates were not significantly different (P = 0.49) to previous nuclear transfer experiments we have conducted with serum starved transgenic and non-transgenic EF5 cells (38%, n = 107; 46%, n = 105; 68%, n = 109; 91%, n = 85; unpublished and ref. [28]).

**Figure 2:**
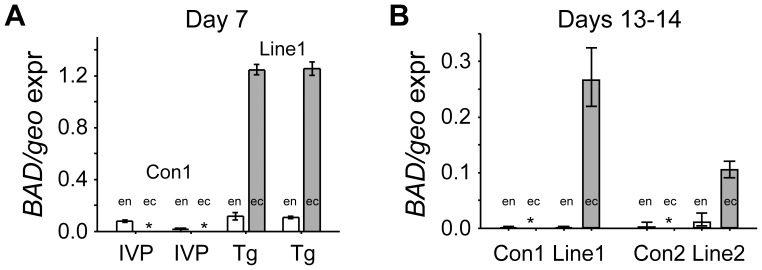
*pCAG-BAD* transgenic cattle embryos express robust levels of *BAD* well in excess of endogenous *BAD* expression. A. Quantitative real-time RT-PCR measurements of two pools (n = 8 embryos) of Day 7 IVP and NT (line 1) embryos transgenic for *BAD*. White bars represent endogenous (*en*) *BAD*, whereas grey bars represent ectopic (*ec*) *BAD* mRNA levels; both normalized against the geomean expression (geo expr) of the three housekeepers *GAPDH*, *Cyclophilin* and *HPRT* (*GAPDH* and *Cyclophilin* levels are generally twice as abundant as the geomean, whereas *HPRT* levels are one fifth). Stars represent non-detectable levels; error bars are s.e.m. B. *BAD* transcript levels for both lines of BAD transgenic Day 13 to 14 embryos and their co-transferred wild type controls. Number of embryos as per Table 3.

Both wild type and transgenic embryos transcribed endogenous *BAD* at low levels (Fig. 2A). However *BAD*-tg embryos expressed ectopic *BAD* abundantly, at similar levels as the geomean of the housekeepers used (Fig. 2A).

**Figure 3:**
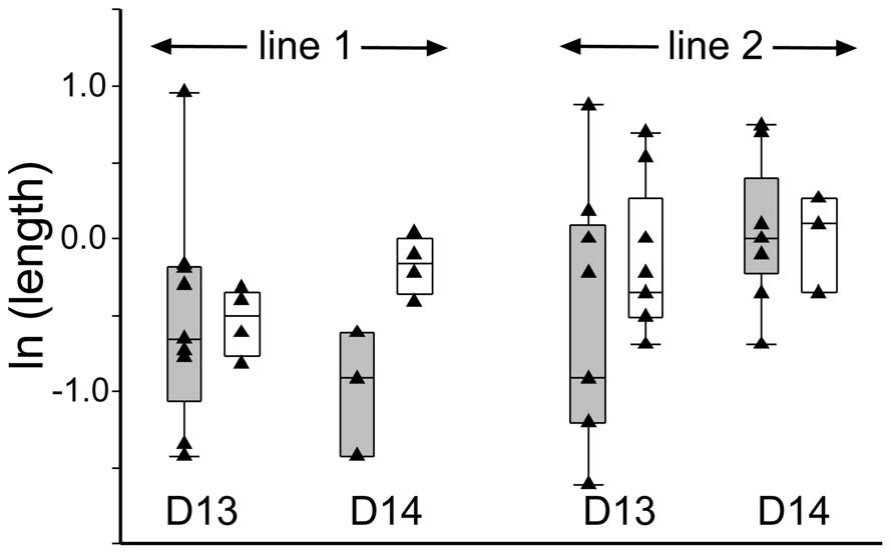
BAD-overexpressing Day 13 and 14 embryos do not differ from control embryos in terms of length. Box and whisker plots depicting median, quartile and 95% values with individual values overlaid for *pCAG-BAD* (grey) and IVP (white) embryos. Distribution of natural logarithm of embryo length in mm is shown for the two individual lines and embryonic day 13 and 14. REML statistical analysis (including recipient effects for the analysis of length) indicated no significant differences.

**Figure 4:**
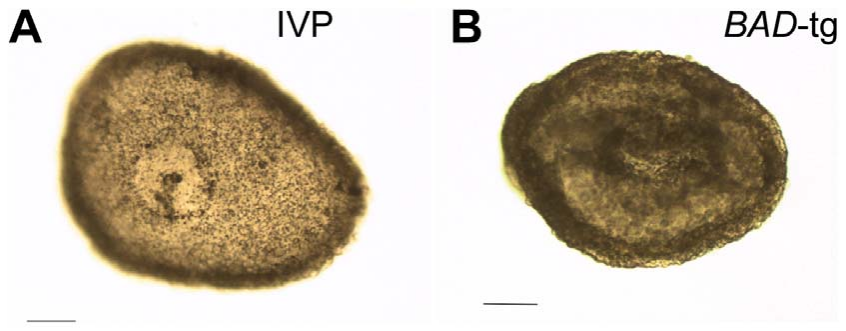
Morphology of *BAD*-transgenic embryos. A. Day 14 IVP embryo with clearly visible embryonic disc. B. Day 14 embryo from *BAD*-transgenic line 2 without a disc and slightly darkened appearance. Bars represent 0.1 mm.

**Figure 5:**
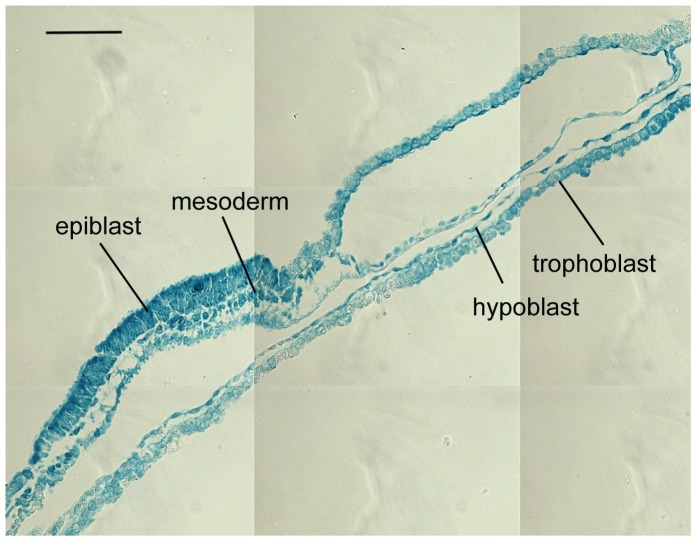
Ubiquitous expression of reporter gene using CAG constructs. Merged sections of an elongation stage Day 15 *CAG-LacZ* transgenic embryo stained for β-Galactosidase. Bar represents 0.1 mm.

We next assessed whether transferable grade *BAD*-tg blastocysts were of equal developmental potential to their non-transgenic in vitro produced counterparts. Transgenic and wild type embryos were transferred into recipient animals and retrieved on Days 13 and 14. At these stages ectopic *BAD* expression had decreased to moderate levels (10–20% of the housekeeper geomean), but were still well in excess of endogenous *BAD* levels (Fig. 2B). From the proportion of embryos recovered, it was clear that continuous *BAD* overexpression had not lead to increased embryo mortality during the second week of development (Table 3). The length of transgenic and non-transgenic embryos did not differ significantly (Fig. 3A). As previously observed, length is highly variable and recipient dependent [8]. However, we saw a striking difference in morphology. A quarter of IVP derived embryos had no epiblast (Table 4), in strict accordance to past observations of IVP [8] as well as nuclear transfer generated embryos [32]. In contrast, 72% of line 1 and all line 2 transgenic embryos were without an embryonic disc/epiblast (Fig. 4, Table 4), a highly significant result. It is unlikely that this is a general nuclear transfer-specific effect based on our previous work using EF5 cells (transgenic and non-transgenic). In those previous experiments, where we also compared co-transferred IVP and nuclear transfer-generated embryos at similar stages, the proportion of embryos without an epiblast was one quarter for all types of embryos [8], [32]. To further verify this, we performed an additional round of NT using an EF5 cell line containing a construct with the LacZ reporter substituting the BAD gene. Nine out of twelve embryos retrieved contained an epiblast (Table 5). We stained one such advanced embryo for β-Gal so as to monitor expression levels in different lineages when using the CAG enhancer/promoter employed for the BAD and LacZ overexpression constructs. Similar expression levels were seen in all lineages (Fig. 5). We thus infer that the loss of the epiblast in BAD transgenic embryos is not a somatic cell transfer or differential expression artifact.

**Table 5:** Significantly fewer *BAD* overexpressing embryos contain an epiblast compared to *LacZ* overexpressing embryos.

	*Embryos retrieved*	*Embryos with Epiblasts (%)*	*P^a^*
pCAG-BAD (line 1)	18	5 (28%)	0.029
pCAG-BAD (line 2)	14	0 (0%)	0.00014
pCAG-LacZ	12	9 (75%)	

*a* Using Fisher's Exact Test.

**Figure 6:**
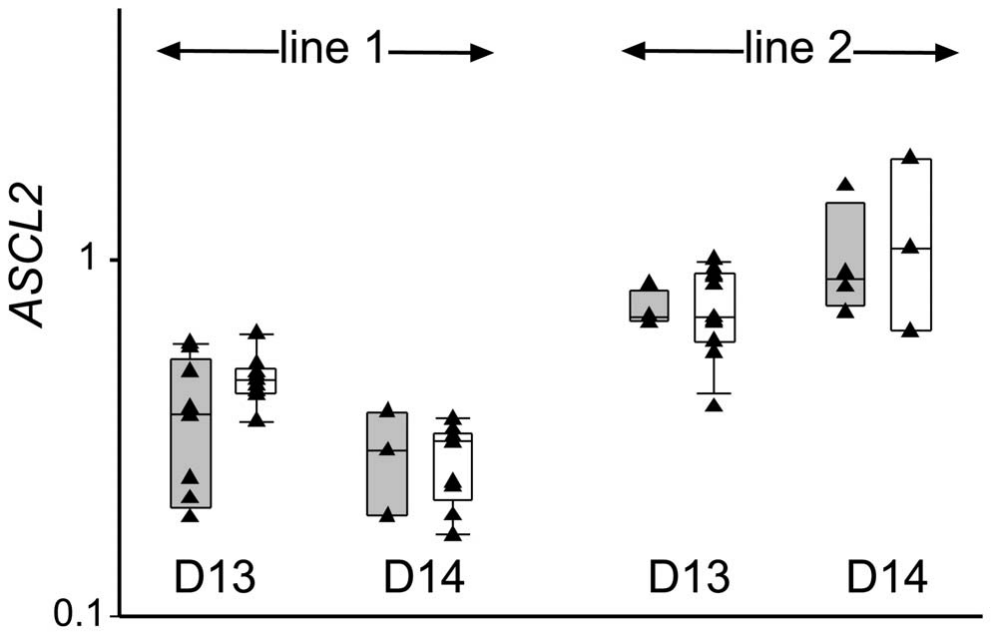
Trophectoderm marker expression. Box and whisker plots depicting median, quartile and 95% values with individual values overlaid for *pCAG-BAD* (grey) and IVP (white) embryos. Distribution of log_10_ of the TE marker *ASCL2*, normalized against three housekeepers, are shown for the two individual lines and embryonic day 13 and 14. REML statistical analysis indicated no significant differences.

We observed that some of the recovered transgenic embryos were slightly darker than their wild type counterparts (example shown in Fig. 4B). This could be caused by a defect in the trophoblast or underlying hypoblast. Embryos were therefore examined for gene expression differences in a TE marker expressed at this stage. *ASCL2* (*Mash2* homolog) is ideally suited for this purpose, as it is expressed maximally in the Day 13 to 14 TE [32]. *ASCL2* was expressed in all transgenic embryos at similar levels to the IVP-derived co-transferred controls (Fig. 6). Overall higher *ASCL2* levels in line 2 embryos appeared to be recipient related as the co-transferred wild type embryos exhibited similar high levels.

**Figure 7:**
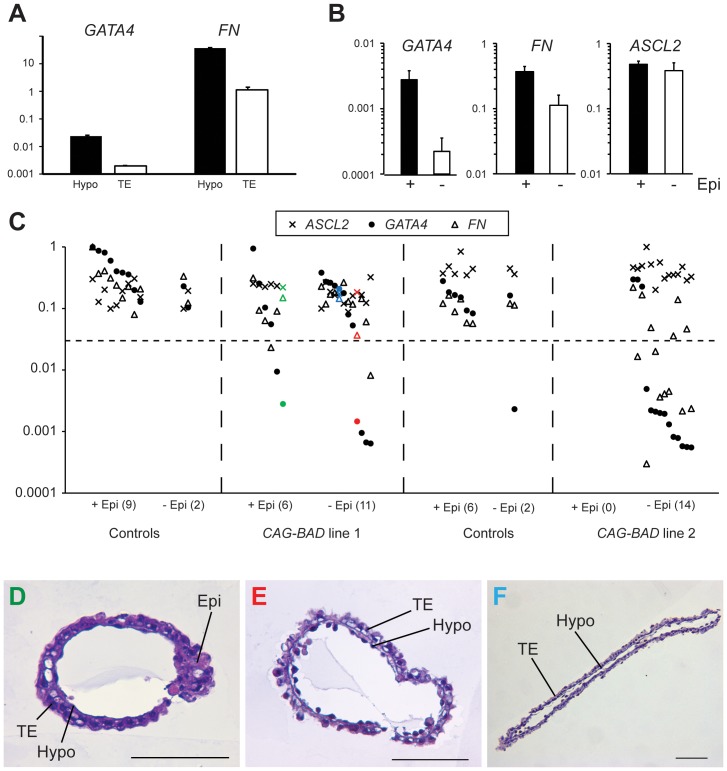
Molecular characterization of BAD-tg embryos for hypoblast and trophectoderm markers. A. Real-time RT-PCR measurement of *GATA4* and *FIBRONECTIN* expression, normalized to the housekeeper *GAPDH*, for isolated Day 14 trophectoderm (TE) and hypoblast (Hypo) tissue. B. Mean *GATA4*, *FIBRONECTIN* and *ASCL2* mRNA levels (normalized to three housekeepers) in epiblast containing (black bar) and epiblast-less embryos. For *GATA4* and *FIBRONECTIN*, P<0.01 (t-test); error bars represent s.e.m. C. *GATA4*, *FIBRONECTIN* and *ASCL2* mRNA levels (log expression for each gene relative to maximum expression value) in trophoblast/hypoblast fragments of *CAG-BAD*-overexpressing and control embryos, clustered into epiblast-containing and epiblast–less groups, with number of embryos per group shown in brackets. Coloured markers represent the three sectioned embryos depicted in panels D-F (coloured correspondingly). D. Cross section of CAG-BAD line 1 embryo containing an epiblast but severely reduced/no *GATA4* expression. E. Cross section of *CAG-BAD* line 1 embryo without an epiblast, severely reduced *GATA4* and lower than average *FIBRONECTIN* expression. F. Embryo as per panel E but showing no altered hypoblast marker expression. Bars are 0.1 mm.

We next investigated whether *BAD* overexpression affected the hypoblast. Wild type Day 14 embryos were treated with proteases to allow mechanical separation of trophoblast (TE) and hypoblast layers. Using these purified cellular preparations, we designed and tested cattle PCR primers for a range of candidate genes based on the mouse literature and our unpublished observations. We determined *GATA4* and *FIBRONECTIN* to be optimal for this purpose with *GATA4* being hardly detectable in the TE and *FIBRONECTIN* showing 40 fold greater expression in the hypoblast (Fig. 7A). Comparing trophoblast + hypoblast tissue of embryos with an embryonic disc to those without revealed highly significant gene expression differences for the two hypoblast markers, but no difference for the trophoblast marker (Fig. 7B). This difference is attributable to the transgenic embryos (Fig. 7C). Most of the BAD-overexpressing line 2 embryos and a third of the line 1 embryos displayed a loss of *GATA4* and reductions in *FIBRONECTIN* expression. This effect on hypoblast marker gene expression was also seen in some BAD overexpressing embryos that did contain an epiblast. The loss/reduction in hypoblast marker gene expression did not reflect a loss of the hypoblast lineage, as determined by sectioning embryos (Fig. 7D–F). We conclude that BAD overexpression leads to changes in hypoblast gene expression but not the loss of this tissue.

## Discussion

We have shown a selective effect of ubiquitous BAD overexpression on the early embryonic lineages. The high frequency of transgenic concepti without epiblast but retaining the hypoblast as well as the TE, indicates that the epiblast is most sensitive to cell death. The epiblast is derived from the ICM which segregates into the epiblast and hypoblast lineages at Day 8, as marked by exclusive expression of NANOG and GATA6 respectively [5]. The survival of the hypoblast under conditions that lead to the loss of the epiblast, indicates firstly that these two ICM derived lineages show differential sensitivity to BAD overexpression. Secondly, one can conclude that the ICM itself does not undergo BAD-induced apoptosis prior to generating the two lineages. While the hypoblast is not lost, its gene expression profile is frequently changed upon BAD overexpression. A loss of GATA4 in hypoblast is likely to have severe developmental consequences based on mouse loss of function studies [36]. On the other hand, FIBRONECTIN secretion by the hypoblast is likely to be required for interactions with the adjacent trophoblast [37].

Notably the trophectoderm is refractory to *BAD* overexpression, as reflected by equal proportions of recoveries of transgenic and wild type embryos and the normal expression of a trophoblast marker characteristic of Day13–14 embryos.

We suggest that the overexpression of BAD, similar to its effects in other systems [25], results in a sensitization of cells to trophic (survival) signals. Hence, in our experiments, the demise of the epiblast indicates insufficient signaling to inhibit the proapoptotic activity of increased BAD protein. BAD can be inactivated by phosphorylation, usually via AKT activation. AKT in turn is activated by PI3K which is the target of multiple receptors that respond to a range of trophic ligands [26]. Interestingly, trophic signals, through AKT activation, not only inactivate BAD but also result in TP53 (p53/Trp53) degradation. This similarly aids cell survival as TP53 enhances apoptosis by activating BAX. Notably, in an in vitro model of early mouse development, TP53 was implicated in a lineage specific effect of culture-induced stress [38]. The authors cultured wild type zygotes for 96 hours in a culture medium deprived of trophic factors. This treatment had previously been shown to reduce the developmental potential of embryos to reach term [39]. When such blastocysts were plated in embryonic stem cell media, their ability to form proliferative ICM/epiblast cultures was much reduced compared to freshly-derived blastocysts. This effect could be partially ameliorated by genetic loss of TP53 and was not seen in corresponding TE outgrowths [38]. The deduction made is that cell culture-induced stress/trophic factor deprivation mediates cell death of ICM derivatives through a failure to prevent TP53 accumulation [17].

Thus activation of either of two branches of the trophic factor-AKT pathway, namely either TP53 or BAD accumulation, leads to a specific loss of ICM derivatives with the epiblast more vulnerable than the hypoblast. Other observations further support the idea that the ICM→epiblast lineage is particularly vulnerable to stress and dependent on survival signals.

1. When attempting to grow cattle embryos past the expanded blastocyst stage in culture, the TE and hypoblast survived well, whereas viable epiblast could not be maintained [11], [12]. This has been interpreted as being caused by a lack of maternal survival signals in the medium.
2. Levels of cell death were higher in the ICM (16–17%) than trophectoderm (3–5%) of both in vitro produced and in vivo-derived cattle blastocysts [40].
3. Approximately one quarter of in vitro produced cattle embryos transferred at the blastocyst stage and retrieved one week later have lost their embryonic disc, with most retaining hypoblast (this study, [8], [41]).
4. Under non-optimal culture conditions, embryonic discs can be lost at even higher rates (50%), indicating the vulnerability of the epiblast [41].

Why is the epiblast most affected? Either (1) the epiblast is exposed to fewer or lower levels of trophic factors and/or (2) it is more dependent on the presence of trophic factors than the TE and hypoblast. In support of (1), the ICM and its derivatives are shielded from the environment, and thus diffusible trophic factors, by the trophectoderm. Only by Day 12 does the polar trophectoderm overlying the epiblast (termed Rauber's Layer in cattle) start to disappear, exposing the epiblast directly to maternal signals which could counteract a proapoptotic effect. In opposition to this line of reasoning is the consideration that the non-differentiated ICM and hypoblast would both suffer similarly from such a TE-shielding effect, yet the hypoblast can survive under conditions where the epiblast does not. Thus, a differential cell survival response to signaling may be the deciding factor. In the mouse, in vitro models exist for all four early lineages. They are trophoblast stem cells (TS) for TE, embryonic stem cells (ES) for ICM/early epiblast, epiblast stem cells (EpiSC) for post-implantation epiblast and extra embryonic endoderm stem cells (XEN) for hypoblast. Of these, EpiSC are the most difficult to maintain, requiring mechanical dissociation for passaging in addition to a range of growth factors [42]. As the epiblast lineage gives rise predominantly to the fetus itself, an increased sensitivity to signals from the environment may allow for the efficient weeding out of suboptimal cells ensuring the generation of a more robust and healthy individual and thus be of selective advantage. Such an early selection process would also be of benefit to the mother, as maintenance of a pregnancy of a developmentally incompetent embryo/fetus is energetically costly.

In conclusion, our model measures, for the first time in an in vivo environment, the importance of survival signaling pathways by raising the threshold of signaling required to overcome deleterious BAD activity. The value of this gain of function approach lies in the insight gained into the inherent differential response of the various lineages to signals which are likely to fluctuate according to environmental conditions. Our results are also relevant in commercial settings. Conceptuses without embryonic disc can survive and develop in the uterus for several weeks and, via IFN-τ secretion, are capable of maintaining the corpus luteum, resulting in a delay in returning to oestrus [43], [44]. Such “non-pregnant” cows are often termed “phantom cows” and, at a prevalence of between 12 and 22% [45] present a serious problem to the pasture-based dairy industry where the seasonal window for pregnancy establishment is small. Embryo loss before Day 28 has been postulated to be a major cause of phantom cows [45]. Our findings highlight that epiblast-susceptibility may underlie a significant fraction of those phantom pregnancies. Our transgenic model may be of practical use in optimizing in vitro embryo culture conditions, by amplifying the beneficial or detrimental effects of media alterations.

## References

[1] Diskin MG, Parr MH, Morris DG (2011) Embryo death in cattle: an update. Reprod Fertil Dev 24: 244–251. 22394965 10.1071/RD11914

[2] Maddox-Hyttel P, Alexopoulos NI, Vajta G, Lewis I, Rogers P, et al (2003) Immunohistochemical and ultrastructural characterization of the initial post-hatching development of bovine embryos. Reproduction 125: 607–623. 12683931

[3] Betteridge KJ, Flechon JE (1988) The anatomy and physiology of pre-attachment bovine embryos. Theriogenology 29: 155–187.

[4] Van Soom A, Boerjan ML, Bols PE, Vanroose G, Lein A, et al (1997) Timing of compaction and inner cell allocation in bovine embryos produced in vivo after superovulation. Biol Reprod 57: 1041–1049. 9369168 10.1095/biolreprod57.5.1041

[5] Kuijk EW, van Tol LT, Van de Velde H, Wubbolts R, Welling M, et al (2012) The roles of FGF and MAP kinase signaling in the segregation of the epiblast and hypoblast cell lineages in bovine and human embryos. Development 139: 871–882. 22278923 10.1242/dev.071688 PMC3274353

[6] Spencer TE, Bazer FW (2004) Conceptus signals for establishment and maintenance of pregnancy. Reprod Biol Endocrinol 2: 49. 15236653 10.1186/1477-7827-2-49 PMC471568

[7] Roche JF, Boland MP, McGeady TA (1981) Reproductive wastage following artificial insemination of heifers. Vet Rec 109: 401–404. 7340073 10.1136/vr.109.18.401

[8] Berg DK, van Leeuwen J, Beaumont S, Berg M, Pfeffer PL (2010) Embryo loss in cattle between Days 7 and 16 of pregnancy. Theriogenology 73: 250–260. 19880168 10.1016/j.theriogenology.2009.09.005

[9] Lane M, Gardner DK (1992) Effect of incubation volume and embryo density on the development and viability of mouse embryos in vitro. Hum Reprod 7: 558–562. 1522203 10.1093/oxfordjournals.humrep.a137690

[10] Gopichandran N, Leese HJ (2006) The effect of paracrine/autocrine interactions on the in vitro culture of bovine preimplantation embryos. Reproduction 131: 269–277. 16452720 10.1530/rep.1.00677

[11] Brandao DO, Maddox-Hyttel P, Lovendahl P, Rumpf R, Stringfellow D, et al.. (2004) Post Hatching Development: A Novel System for Extended In Vitro Culture of Bovine Embryos. Biology of Reproduction. 10.1095/biolreprod.103.025916 15329327

[12] Vajta G, Alexopoulos NI, Callesen H (2004) Rapid growth and elongation of bovine blastocysts in vitro in a three-dimensional gel system. Theriogenology 62: 1253–1263. 15325552 10.1016/j.theriogenology.2004.01.007

[13] Gray CA, Taylor KM, Ramsey WS, Hill JR, Bazer FW, et al (2001) Endometrial glands are required for preimplantation conceptus elongation and survival. Biol Reprod 64: 1608–1613. 11369585 10.1095/biolreprod64.6.1608

[14] Loureiro B, Oliveira LJ, Favoreto MG, Hansen PJ (2011) Colony-stimulating factor 2 inhibits induction of apoptosis in the bovine preimplantation embryo. Am J Reprod Immunol 65: 578–588. 21223422 10.1111/j.1600-0897.2010.00953.x

[15] Byrne AT, Southgate J, Brison DR, Leese HJ (2002) Regulation of apoptosis in the bovine blastocyst by insulin and the insulin-like growth factor (IGF) superfamily. Mol Reprod Dev 62: 489–495. 12112582 10.1002/mrd.10153

[16] Lu DP, Chandrakanthan V, Cahana A, Ishii S, O'Neill C (2004) Trophic signals acting via phosphatidylinositol-3 kinase are required for normal pre-implantation mouse embryo development. J Cell Sci 117: 1567–1576. 15020683 10.1242/jcs.00991

[17] O'Neill C, Li Y, Jin XL (2012) Survival signaling in the preimplantation embryo. Theriogenology 77: 773–784. 22325248 10.1016/j.theriogenology.2011.12.016

[18] Bedzhov I, Liszewska E, Kanzler B, Stemmler MP (2012) Igf1r Signaling Is Indispensable for Preimplantation Development and Is Activated via a Novel Function of E-cadherin. PLoS Genet 8: e1002609. 22479204 10.1371/journal.pgen.1002609 PMC3315466

[19] Makarevich AV, Markkula M (2002) Apoptosis and cell proliferation potential of bovine embryos stimulated with insulin-like growth factor I during in vitro maturation and culture. Biol Reprod 66: 386–392. 11804953 10.1095/biolreprod66.2.386

[20] Loureiro B, Bonilla L, Block J, Fear JM, Bonilla AQ, et al (2009) Colony-stimulating factor 2 (CSF-2) improves development and posttransfer survival of bovine embryos produced in vitro. Endocrinology 150: 5046–5054. 19797121 10.1210/en.2009-0481 PMC2775977

[21] Yang E, Zha J, Jockel J, Boise LH, Thompson CB, et al (1995) Bad, a heterodimeric partner for Bcl-XL and Bcl-2, displaces Bax and promotes cell death. Cell 80: 285–291. 7834748 10.1016/0092-8674(95)90411-5

[22] Zha J, Harada H, Yang E, Jockel J, Korsmeyer SJ (1996) Serine phosphorylation of death agonist BAD in response to survival factor results in binding to 14-3-3 not BCL-X(L). Cell 87: 619–628. 8929531 10.1016/s0092-8674(00)81382-3

[23] Lindsten T, Ross AJ, King A, Zong WX, Rathmell JC, et al (2000) The combined functions of proapoptotic Bcl-2 family members bak and bax are essential for normal development of multiple tissues. Mol Cell 6: 1389–1399. 11163212 10.1016/s1097-2765(00)00136-2 PMC3057227

[24] Ranger AM, Zha J, Harada H, Datta SR, Danial NN, et al (2003) Bad-deficient mice develop diffuse large B cell lymphoma. Proc Natl Acad Sci U S A 100: 9324–9329. 12876200 10.1073/pnas.1533446100 PMC170917

[25] Datta SR, Ranger AM, Lin MZ, Sturgill JF, Ma YC, et al (2002) Survival factor-mediated BAD phosphorylation raises the mitochondrial threshold for apoptosis. Dev Cell 3: 631–643. 12431371 10.1016/s1534-5807(02)00326-x

[26] Danial NN (2008) BAD: undertaker by night, candyman by day. Oncogene 27 Suppl 1S53–70. 19641507 10.1038/onc.2009.44

[27] Chambers I, Colby D, Robertson M, Nichols J, Lee S, et al (2003) Functional expression cloning of Nanog, a pluripotency sustaining factor in embryonic stem cells. Cell 113: 643–655. 12787505 10.1016/s0092-8674(03)00392-1

[28] Berg DK, Smith CS, Pearton DJ, Wells DN, Broadhurst R, et al (2011) Trophectoderm lineage determination in cattle. Dev Cell 20: 244–255. 21316591 10.1016/j.devcel.2011.01.003

[29] Oback B, Wells DN (2003) Cloning cattle. Cloning Stem Cells 5: 243–256. 14733744 10.1089/153623003772032763

[30] Thompson JG, McNaughton C, Gasparrini B, McGowan LT, Tervit HR (2000) Effect of inhibitors and uncouplers of oxidative phosphorylation during compaction and blastulation of bovine embryos cultured in vitro. J Reprod Fertil 118: 47–55. 10793625

[31] Robertson I, Nelson R (1998) Certification and identification of the embryo. In: Stringfellow DA, Seidel SM, editors. Manual of the International Embryo Transfer Society: (International Embryo Transfer Society). pp. pp. 103–134.

[32] Smith CS, Berg DK, Berg M, Pfeffer PL (2010) Nuclear transfer-specific defects are not apparent during the second week of embryogenesis in cattle. Cell Reprogram 12: 699–707. 20973678 10.1089/cell.2010.0040

[33] Donnison M, Beaton A, Davey HW, Broadhurst R, L'Huillier P, et al (2005) Loss of the extraembryonic ectoderm in Elf5 mutants leads to defects in embryonic patterning. Development 132: 2299–2308. 15829518 10.1242/dev.01819

[34] Smith C, Berg D, Beaumont S, Standley NT, Wells DN, et al (2007) Simultaneous Gene Quantitation of Multiple Genes in Individual Bovine Nuclear Transfer Blastocysts. Reproduction 133: 231–242. 17244749 10.1530/rep.1.0966

[35] Youle RJ, Strasser A (2008) The BCL-2 protein family: opposing activities that mediate cell death. Nat Rev Mol Cell Biol 9: 47–59. 18097445 10.1038/nrm2308

[36] Narita N, Bielinska M, Wilson DB (1997) Wild-type endoderm abrogates the ventral developmental defects associated with GATA-4 deficiency in the mouse. Dev Biol 189: 270–274. 9299119 10.1006/dbio.1997.8684

[37] Takahashi M, Takahashi M, Hamano S, Takahashi H, Okano A (2005) In vitro attachment of bovine hatched blastocysts on fibronectin is mediated by integrin in a RGD dependent manner. J Reprod Dev 51: 47–57. 15750296 10.1262/jrd.51.47

[38] Ganeshan L, Li A, O'Neill C (2010) Transformation-related protein 53 expression in the early mouse embryo compromises preimplantation embryonic development by preventing the formation of a proliferating inner cell mass. Biol Reprod 83: 958–964. 20739669 10.1095/biolreprod.109.083162

[39] Li A, Chandrakanthan V, Chami O, O'Neill C (2007) Culture of zygotes increases TRP53 [corrected] expression in B6 mouse embryos, which reduces embryo viability. Biol Reprod 76: 362–367. 17093197 10.1095/biolreprod.106.056838

[40] Leidenfrost S, Boelhauve M, Reichenbach M, Gungor T, Reichenbach HD, et al (2011) Cell arrest and cell death in mammalian preimplantation development: lessons from the bovine model. PLoS One 6: e22121. 21811561 10.1371/journal.pone.0022121 PMC3141016

[41] Fischer-Brown AE, Lindsey BR, Ireland FA, Northey DL, Monson RL, et al (2004) Embryonic disc development and subsequent viability of cattle embryos following culture in two media under two oxygen concentrations. Reprod Fertil Dev 16: 787–793. 15740702 10.1071/rd04026

[42] Nichols J, Smith A (2011) The origin and identity of embryonic stem cells. Development 138: 3–8. 21138972 10.1242/dev.050831

[43] Heyman Y, Camous S, Fevre J, Meziou W, Martal J (1984) Maintenance of the corpus luteum after uterine transfer of trophoblastic vesicles to cyclic cows and ewes. J Reprod Fertil 70: 533–540. 6699815 10.1530/jrf.0.0700533

[44] Nagai K, Sata R, Takahashi H, Okano A, Kawashima C, et al (2009) Production of trophoblastic vesicles derived from Day 7 and 8 blastocysts of in vitro origin and the effect of intrauterine transfer on the interestrous intervals in Japanese black heifers. J Reprod Dev 55: 454–459. 19420837 10.1262/jrd.20222

[45] Cavalieri J (2003) Phantom cows: predisposing factors, causes and treatment strategies that have been attempted to reduce the prevalence within herds. Veterinary Continuing Education (Proceedings of Australian and New Zealand Combined Dairy Veterinarians Conference) 227: 365–388.

